# Liberia's Community Health Assistant Program: Scale, Quality, and Resilience

**DOI:** 10.9745/GHSP-D-20-00509

**Published:** 2021-03-15

**Authors:** Jessica Healey, S. Olasford Wiah, Jannie M. Horace, Dianah B. Majekodunmi, Derry S. Duokie

**Affiliations:** a Liberia Health Office, U.S. Agency for International Development, Monrovia, Liberia.; b Community Health Services Division, Ministry of Health, Monrovia, Liberia.; c International Rescue Committee, Monrovia, Liberia.

## Abstract

Liberia's community health program went from concept to nationwide scale in 4 years due to the Liberian Government's vision and its partnership with implementing organizations and donors. The next community health policy will tackle the unfinished agenda related to quality, resilience, and sustainability. Liberia's experience offers valuable lessons for innovating, and institutionalizing a compensated, effective cadre of community health assistants.

## Key Messages

- Liberia's national community health program went from dispersed pilots to nationwide scale within 4 years.
- A network of policy entrepreneurs from the Liberian government, donors, and implementing partners capitalized on several windows of opportunity to achieve this success.
  - First, they seized the evidence from a series of pilot projects and—during a point of significant global momentum around community health—evaluated the agenda.
  - Second, they leveraged the impact of the community health workers during the Ebola virus disease outbreak to propel forward a consolidated, paid cadre with significant political and financial backing.
- Policy makers and program managers should consider coalition building and identifying diverse champions as an essential ingredient for impacting large-scale change, even in the face of significant challenges.
- Liberia's community health program is bolstered by significant research and evidence. However, the policy entrepreneurs who brought the community leaders, Government of Liberia stakeholders, donors, and partners around the table to buy into a common program has been the “secret sauce” of the program's success.

## INTRODUCTION

Libera has weathered significant financial, economic, and social hardship including over a decade of civil war and a year-long outbreak of Ebola virus disease (EVD). Over half of the country lives in poverty. The country's economic situation has been challenged by limited agricultural production and reliance on exports of raw materials, subject to global fluctuations in value. Given these challenges, how did Liberia transition from small-scale community health pilot programs to a government-led nationwide community health assistant (CHA) program in just 4 years?

We argue that this success and the emergence of the community health program as a central pillar of Liberia's health strategy can be attributed to the convergence of ideas, interests, and institutions during significant windows of opportunity in Liberia. This catalytic and unifying change was driven by networks of “policy entrepreneurs”^1^^,^^2^ many of whom—in the Liberian government, among donors, and implementing partners—continue to guide and refine the strategy and program.

An important opportunity presented itself after community health workers were successfully mobilized to respond to the EVD outbreak of 2014–2016, thrusting the community program onto “center stage” in Liberia. During this period, community-based information systems for infectious disease surveillance and response were strengthened and integrated into the community health program. Leaders within the Ministry of Health (MOH), as well as funding and implementing agencies, capitalized on this momentum that led to the harmonized expansion of the program and contributes to the country's ability to respond to the coronavirus disease (COVID-19) pandemic and other health threats as they arise.

## THE NATIONAL COMMUNITY HEALTH ASSISTANT PROGRAM

CHAs are a formal, standardized, and compensated cadre of community health providers. They are supported by community health services supervisors within the community health structures such as community health committees. The CHAs serve as the backbone of the revised national community health services policy and plan for 2016–2021,^3^ which aims to extend the reach of the country's primary health care system to provide a package of essential lifesaving primary health care services and epidemic surveillance within communities and to households on an equitable basis. CHAs—literate men and women who live in the communities they serve—are selected by their respective communities. They receive training to deliver an integrated and standardized service delivery package, which includes promotive, preventive, and curative services and epidemic surveillance, to households located more than 5 km from the nearest health facility (estimated at 29% of the population as of the last census). CHAs provide health education; test for and treat malaria; provide family planning methods, oral rehydration solution, and zinc; refer pregnant women for antenatal care; assist in mass immunization campaigns; and oversee mass drug administration. Community event-based surveillance of infectious diseases became an important component of CHA training and responsibilities during the EVD outbreak and provided a strong platform for addressing the COVID-19 pandemic response.

By early 2020, the CHA program covered 80% of all communities outside of the 5 km radius of a health facility in 14 of the 15 counties of Liberia.^4^ The national report for 2019^5^ highlighted the following achievements in the top priority indicators (Table). By June 2021, the CHA program is expected to achieve full county coverage of all targeted communities in all 15 counties. At the time of writing, the program has 3,448 CHAs (2,862 male [83%] and 586 female [17%] and 373 community health services supervisors (191 male [51%] and 182 female [49%]). CHAs have not only expanded the reach of primary care into remote villages but have also contributed to improvements in maternal and child health, as reflected in the 2019/2020 Liberia Demographic and Health Survey that noted a considerable increase in skilled birth attendance and facility deliveries.^6^

**TABLE.** Achievements of the Community Health Assistants in Liberia, by Indicator, 2019^a^

	Target	Achievement
Children under 5 years of age who tested positive for malaria and treated with artemisinin-based combination therapy within 24 hours, %	60	57.7
Children under 5 years of age who were diagnosed with pneumonia and treated with antibiotics, %	91	87.6
Children under 5 years of age who were treated for diarrhea with oral rehydration solution and zinc, %	87	83.3
Cases treated with appropriate medicine and dose (as confirmed by CHSS during supervision visits), %	95	87.4
Pregnant women referred for delivery, no.	22,792	19,907^b^

Abbreviations: CHA, community health assistant; CHSS, community health services supervisor.

a Reporting rate for community health assistant was 91%.

b 42% of the total expected pregnant women in the community health assistant population area.

The CHA program, established in 2016, is expected to achieve 100% coverage of all targeted communities by June 2021.

For its achievements to revitalize primary health care through the national community health program, Liberia has garnered global recognition,^7^ including being nominated to host the next Global Community Health Symposium. Liberia's new National Community Health Services Policy (2021–2027) is poised to tackle the unfinished agenda focusing on national scale, quality, resilience, and sustainability. Liberia's ambitions and experiences in adapting, innovating, and institutionalizing the national CHA program at national scale can serve as valuable lessons for countries in the midst of boldly redefining and reforming their community health platforms.

## FACTORS CONTRIBUTING TO CHA PROGRAM SCALE AND SCOPE

The CHA program's success was determined by 3 major factors: (1) foundational success with community volunteers through a series of pilots coupled with global momentum, presenting an opportunity for consolidation of policy and programs; (2) emergence of community health workers as the champions of the EVD crisis, presenting another opportunity for large-scale change; and (3) significant leadership from policy entrepreneurs at the community to executive levels who drove change and propelled unity of focus and purpose.

### 1. Foundational Success With Community Health Volunteers and Global Momentum

Like many countries, Liberia's initial focus in community health was largely on malaria, pneumonia, and acute respiratory illness with unpaid volunteers, called general community health volunteers (gCHVs). The implementation was through various models supported by different partners, some that aligned with the 2011–2015 policy^8^ and focused more squarely on integrated community case management (iCCM) and some that explicitly went beyond the bounds of the current policy (e.g., offered remuneration for gCHVs).^9^ These pilots produced largely robust, positive results and demonstrated that lay community members with basic training and supervision could deliver information, testing, and referrals for malaria, pneumonia, and diarrhea.

**Figure:**
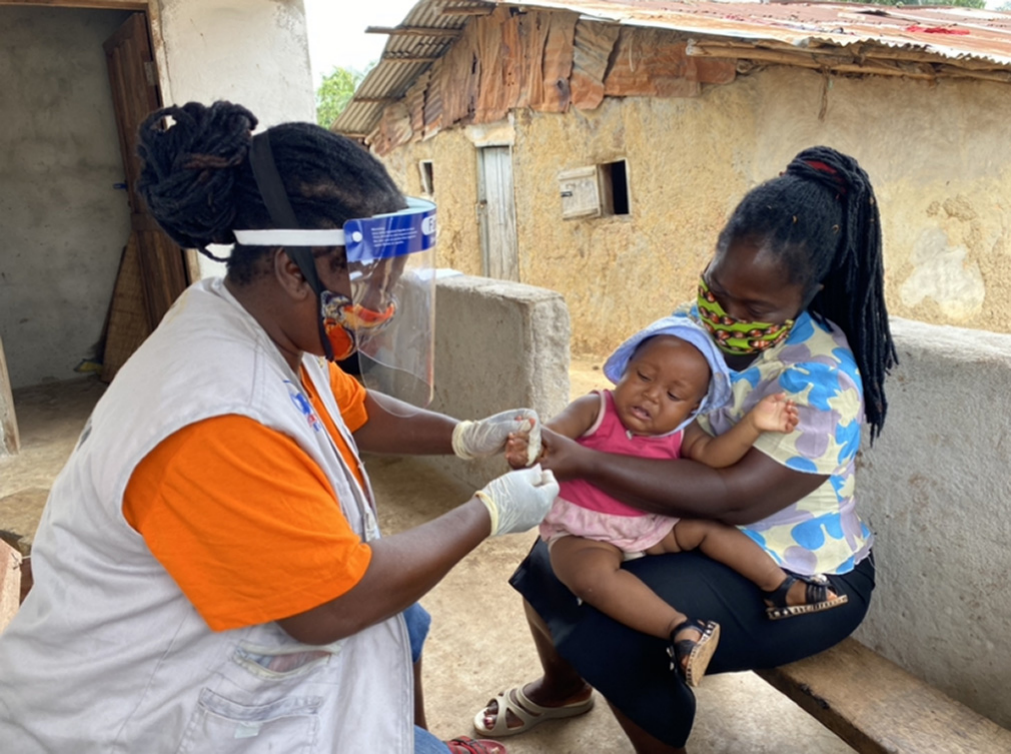
A community health assistant in Suakoko, Bong County, Liberia, conducts a rapid diagnostic test for malaria on a child. © 2020 Jefyne Togba/International Rescue Committee

The Minister of Health at the time, the Honorable Dr. Walter T. Gwenegale, visited a remote pilot site in Grand Gedeh County and contributed to the momentum and leadership behind the 2010 version of the community health policy that included a harmonized iCCM training, gCHV treatment registers, and the initial tools for the community health information system. As early as 2011, Dr. Gwenegale coauthored an article suggesting that^10^:

*a substantial rural health delivery gap remains, but it could be bridged with a robust cadre of community health workers integrated into the primary health care system.*

Building on this early program infrastructure, 4 partners (Africare, EQUIP, International Rescue Committee, and Child Fund) received MOH approval for their proposals to expand the pilot phase in Gbarpolu, Lofa, Nimba, and Bong counties. The pilot trained 114 gCHVs in 93 catchment communities. These iCCM pilots laid a foundation to develop a scalable model for engaging communities in primary health care systems through strengthening and reestablishing community governance structures (such as the community health committees) and the introduction of a community health workforce (Box 1). The pilots also revealed key challenges and deficiencies in design that influenced the future policy. Some of these critical challenges were related to training length and scope, incentives and tools, supply chain, and health systems functions and reporting.

BOX 1 **Spotlight on Current Director of the Ministry of Health Community Health Services Division**

Mr. S. Olasford Wiah, co-author and Director of the Ministry of Health Community Health Services Division, recalled a period when he was the HIV/TB focal person in River Gee County as the turning point in his career. One of his patients with HIV, who lived in a remote village and to whom he was particularly close, died along with her baby after prolonged labor. At that time and during that period of grief, he learned about the integrated community case management pilots and was appointed as County Community Health Department Director. That is when he decided to focus on community health and “do all it takes to strengthen local community structures.” He noted, “I strongly believe that if there was a community health worker in that village, my patient would be alive today.” Mr. Wiah has since been a driving force behind the scale-up of the community health assistant program.

The iCCM pilots laid a foundation to develop a scalable model for engaging communities in primary health care systems through strengthening and reestablishing community governance structures.

In 2015, Liberia finalized its Community Health Roadmap, which was an important coalition building process in its own right, bringing in 100 participants from 50 organizations, government, and nongovernmental programs, including the MOH departments, county health team representatives, United Nations agencies, bilateral and donor agencies, and local and international nongovernmental organizations. The roadmap began addressing the major components of the future policy, including standardized incentives, a draft service package, and integration across other systems, and paved the way for many of the “policy entrepreneurs” to further network and solidify ideas and aspirations for the program.

This early work built on global momentum to prioritize community health emanating from the global campaign, led by Jeffrey Sachs, for 1 million community health workers by 2015, which was helpful for bringing the international community squarely to the table.^11^

### 2. Emergence of Paid Community Health Workers as the Champions of the EVD Crisis

By the height of the EVD epidemic in 2014, the MOH changed its strategy from a largely top-down, directive approach that was not achieving results to a bottom-up approach, engaging more with the communities to listen and find solutions. The approach utilized the governance system at the subnational level to consult with local leaders including chiefs, traditional leaders, and other community groups and employed multimedia strategies to address traditional practices that were putting people at risk.

Although development partners came to consensus on how to engage communities through local structures and gCHVs, there was no common guidance on tools and incentives. Development partners introduced incentives at varying amounts to social mobilizers, community engagement officers, and contact tracers. By the end of the outbreak, it became clear that with an appropriate motivation package and adequate supervision, CHVs could deliver quality services. It also became clear that the various proposed incentives, tools, and approaches were creating inflated expectations and some chaos within the community health program. It was widely seen as not feasible to revert to a voluntary system or one with ad hoc, limited supervision.

The success of CHVs who supported social mobilization, community engagement, and contact tracing during the EVD crisis were lauded at the highest levels. In her remarks at a U.S. Senate Foreign Relations Subcommittee Hearing, President Ellen Johnson Sirleaf stated^12^:

*I could not agree more about building local capacity. Our 10-year health workforce plan is about building capacity at all levels, particularly at the bottom. It's like a pyramid. We will train … community health workers to provide basic services … we are going to make the final push to fight Ebola now by supporting community workers to get the job done.*

This support provided an opening for the new community health program and CHAs to be institutionalized, with common training, deployment tools, and supervision structures (Box 2).

BOX 2 **Spotlight on Previous Ministry of Health Leadership**

Dr. Bernice T. Dahn, in her role as chief medical officer and Deputy Minister for Health Services, was another driving force behind the consolidation of many community health volunteer programs into the community health assistant program. During the early stages of the Ebola virus disease outbreak in 2014–2015, Dr. Dahn coordinated the national response to the epidemic. Upon establishment of the incident management system, she focused on community-based initiatives and restoration of routine health care services. Community health services was at the center of this effort to mobilize communities to restore and rebuild trust in the health system. Under the leadership of Mr. Tamba Boima, Director of the Ministry of Health (MOH) Community Health Services at the time, the MOH led several community engagement and dialogue sessions with community leaders and catchment communities and service providers. These sessions informed national stakeholders about the many resources and solutions available at the community level and provided lessons on how to promote partnerships with these communities for future outbreaks.

By the close of 2015, the previous policy was coming to an end. After a global review, the MOH arrived at paying the CHAs US$70 monthly. The new National Community Health Services Strategic Plan 2016–2021 institutionalized the CHAs and their community health services supervisors.^3^ As the EVD outbreak ended, a new policy was launched, reflecting Liberia's vision for a stronger role for communities in the primary health care system by including paid and supervised CHAs, community governance structures, and a community-based information system.^13^

The new community health policy was launched, reflecting Liberia's vision for a stronger community role in the primary health care system by including paid and supervised CHAs.

### 3. Strong Leadership From Policy Entrepreneurs

Without the unrelenting leadership from communities to the highest levels of the national government, the national CHA program could not have been harmonized, scaled up, and continually adapted for quality and sustainability through changing administrations. During the pilot process and earlier periods, Mr. Tamba Boima, Director of the MOH Community Health Services Division during the EVD outbreak, catalyzed early successes of the pilot and built on the political leadership of Liberia's Minister of Health and President to bring the technical components and partners together in a common plan. By highlighting community health as a flagship program, President Sirleaf paved the way for significant MOH support from donors and development partners.

As the program was developing, the MOH-led orientation and advocacy meetings with heads of ministries and agencies (ministries of youth and sports, internal affairs, gender, and child protection, etc.), county superintendents, and district commissioners gained widespread buy-in from political and traditional leaders. This leadership continues to bring diverse partners together, providing a platform for focus, financial, and technical support. These partners included U.S. Agency for International Development (USAID), the World Bank, Last Mile Health, United Nations Children's Fund (UNICEF), Partners In Health, Samaritan's Purse, Co-Impact, and the Global Fund. The significance of this number of funding partners and the investment in a common program cannot be overstated.

Although there were central figures who drove the overall program implementation, the leadership across the MOH from national to subnational levels that pursued the intentional integration of core health services and systems with the community health program was equally important and likely bolstered the program's longevity and resilience in the face of political and country changes. For example, the new community health program was integrated into human resource guidelines, information systems, supply chain protocols, and the country's research agenda. The MOH leaders' actions to intentionally integrate the program created more government ownership of the program, made it less of a temporary “donor” program, and made the program more resilient to future changes.

MOH leadership across all levels that sought integration of core health services and systems into the community health program was important and likely bolstered the program's longevity and resilience in the face of political and country changes.

The current Director of the MOH Community Health Services Division, S. Olasford Wiah, has maintained this momentum and focus by conducting routine reviews, forming a MOH-led steering committee, and developing a new “One County, One Partner” 2020 plan* that ensures each county's community health program is evenly supported. He has also prioritized adequate time and investment in research and adaptation of the CHA program over time.

This strong leadership was particularly important through the scale-up of the national CHA program. The MOH's persistent engagement with donors and direction through the One County, One Partner plan enabled gradual scale-up in 14 of the 15 counties toward full coverage.

## FUTURE FOR COMMUNITY HEALTH IN LIBERIA

Across Liberia, CHAs and CHVs play a pivotal role in supporting the national health system and now the COVID-19 response effort, especially in rural communities and in intercounty border communities where access to facility-based health services is limited. The dynamic partnership between health donors and the MOH to support a truly nationwide, harmonized, national CHA program steered by strong leadership and regular reviews brings health closer to the Liberian people. Today, the CHA program is widely viewed as a realistic entry point for other innovative work being introduced in Liberia including Sayana Press, a uniject family planning method, mental health services, and treatment for severe malaria among young children before referral.

The CHA program is widely viewed as a realistic entry point for other innovative work being introduced in Liberia.

Nevertheless, significant challenges remain. Variations between implementation in different counties due to different partners remains, despite having a harmonized plan and policy. The supply chain challenges that plague the overall health system also impact commodities and supplies for CHAs and represent a significant bottleneck to overall implementation. Supervision structures are frequently weak, which hampers the quality of care provided. Routine changes with payment and support for the CHAs due to funding gaps or other challenges continue.^5^

One significant future challenge is that the CHA program depends almost entirely on donor funding. Even though some salary support from the government is in place, the program cannot provide sufficient training and supervision. The program has held together and grown stronger through several different donor funding cycles. The World Bank, UNICEF, USAID, and the Global Fund all went through new strategy processes and made new funding decisions within the past year; all maintained or increased their support for the CHA program.

However, this external support is not a guarantee. Stakeholders and the MOH routinely express a need to increase domestic funding to ensure the sustainability of this important program, but with Liberia's limited fiscal space, it is unlikely this is on the immediate horizon. Liberia will have to think creatively about sustainability beyond full financial coverage. This could involve graduating communities from the program that have reached a certain level of program achievement or integrating resource mobilization strategies into the CHA program.

Another challenge is the harmonization and integration of the gCHV program for service delivery within 5 km into the overall national strategic plan. More than a third of Liberia's population lives in urban centers that have different but significant health needs. The MOH is advocating for a CHA approach within these urban centers to address the needs and maximize the efficient use of resources for a clearly defined, singular program.

Critical to overcoming these challenges is renewing and expanding political support across the government and down to the community level for the CHA program, particularly as leadership changes. Some of the new political leadership is not aware of the long history and buy-in for the community health program and see it as a “donor activity.” Without strong domestic political support, the national CHA Program is in a precarious position to garner greater financial support from the Government of Liberia and to maintain donor commitment.

Critical to overcoming the future challenges to sustaining the CHA program's success is renewing and expanding political support across the government and down to the community level.

In November 2021, Liberia will host the next Global Community Health Symposium. The MOH, its partners, and supporters are planning internal advocacy in advance of the symposium to improve this buy-in and rekindle the broad commitment. This provides an important opportunity for Liberia to share its lessons learned with other countries and to gain insights from other country programs. It also provides the program in Liberia with an important opportunity to further bolster the political engagement, particularly among leaders outside the MOH, with the CHA concept and the critical role the community health program plays in the health sector.

Ebola and then COVID-19 illustrated the power and resilience of frontline workers to adapt in the face of new needs and challenges. New diseases will continue to plague both Liberia and the world. All eyes must remain on those community-based workers as the foundation of our health system for maternal and child health services and for epidemic preparedness.

## Footnotes

* This leadership brought Global Fund onboard to support Bomi, Margibi, and additional areas of Lofa and Nimba. With the new “one county, one partner strategy,” the World Bank will support Gbarpolu and Grand Cape Mount; Last Mile Health will continue in Rivercess and expand in Grand Bassa. Global Fund will expand in Nimba, Margi, Montserrado, and Bomi. USAID will expand in Lofa and Bong by 2021. At the time of writing, the support for Grand Gedeh, River Gee, Maryland, Grand Kru, and Sinoe is under discussion.

## Notes

**Peer Reviewed**
